# Aujeszky’s disease in hunting dogs after the ingestion of wild boar raw meat in Sicily (Italy): clinical, diagnostic and phylogenetic features

**DOI:** 10.1186/s12917-022-03138-2

**Published:** 2022-01-08

**Authors:** Flavia Pruiti Ciarello, Ana Moreno, Nicola Miragliotta, Aliberti Antonino, Michele Fiasconaro, Giuseppa Purpari, Benedetta Amato, Dorotea Ippolito, Vincenzo Di Marco Lo Presti

**Affiliations:** 1 Istituto Zooprofilattico Sperimentale della Sicilia “A. Mirri”, Via Gino Marinuzzi, 3, 90129 Palermo, Italy; 2 National Reference Center for Aujeszky’s Disease, Istituto Zooprofilattico Sperimentale Della Lombardia E Dell’Emilia-Romagna “ Bruno Ubertini”, Via Bianchi, 9 - 25124 Brescia, Italy

**Keywords:** Aujeszky, SuHV-1, Interspecies transmission, Hunting dogs, Wild boar, Sicily, Italy

## Abstract

**Background:**

Aujeszky's disease is caused by Suid Herpes Virus-1 and species belonging to the genus *Sus scrofa* are the main reservoir hosts. This virus, however, is capable of infecting and causing severe disease, with an almost constant fatal outcome in other species, both domestic and wild (carnivores, monogastric herbivores and ruminants). Moreover, the possibility of transmission to humans has been demonstrated. This study reports and describes the clinical, diagnostic, pathological and phylogenetic aspects of two cases of Aujeszky's disease in two hunting dogs following the ingestion of infected wild boar raw meat. These cases are contextualized in the province of Messina (Sicily), where a high prevalence of Aujeszky's disease has been recorded (average of 12,20% in the period 2010–2019) in farmed pig, and with evidence of spread to other species. A severe outbreak in cattle has recently been reported in these areas. Nevertheless, cases of Aujeszky's disease in dogs are rarely reported and this study represents the first well-documented report in this species in Sicily.

**Case presentation:**

After a wild boar hunt, two dogs showed neurological symptoms and intense itching unresponsive to therapy. Diagnosis of Aujeszky's disease was made based on clinical suspicion, anamnestic information and confirmed by the isolation of the virus from the brain of both dogs. In addition, molecular typing, sequencing and phylogenetic analysis of the Real-Time PCR products were performed. The sequences studied were placed in the Italian Clade 1 along with the sequences obtained from wild boars and hunting dogs from Italy and France.

**Conclusions:**

The finding of this disease in non-natural hosts in Sicilian multi-host epidemiological contexts suggests that the risk of inter-species transmission is concrete and that attention should be paid to developing disease control programs in these territories. The data obtained from genome sequencing of the two SuHV-1 isolates contribute to the enrichment of the GenBank with unknown sequences and the phylogenetic analysis implementation.

**Supplementary Information:**

The online version contains supplementary material available at 10.1186/s12917-022-03138-2.

## Background

Aujeszky's disease (AD), commonly known as Pseudorabies, is a contagious viral disease caused by Suid Herpes Virus 1 (SuHV-1), which belongs to the Herpesviridae family, subfamily Alphaherpesvirinae, genus *Varicellovirus* [1]. The reservoir hosts of AD are domestic/feral pigs [2] and wild boars [3–8]. In these species indeed, SuHV-1 goes into latency, persisting in nerve ganglia, with the possibility of viral reactivation and consequent excretion and transmission. In pigs, the severity of neurological, respiratory and reproductive symptoms decreases with age, reflecting a greater efficiency of the immune system in adults than in young pigs [9]. On the other hand, in the European wild boar, strains with low virulence and a lower pathogenic potential compared to the domestic pig have been identified [10], and manifest clinical cases have been reported only sporadically in Europe [11, 12]. However, SuHV-1 can infect and cause severe disease in ruminants, carnivores, rodents, horses and lagomorphs, often characterized by the presence of intense itching with an almost constantly lethal outcome [9, 13–16]. Pruritus, almost constant in non-porcine species, is rare in pigs [17, 18]. The recent confirmation of SuHV-1 infections reported in humans with endophthalmitis and/or encephalitis [19, 20] indicates that SuHV-1 is also a potential threat for humans, especially in categories (e.g. veterinarians and animal management personnel) exposed to contact with potentially infected pigs, cattle and carnivores [19, 21–27]. Clinical cases of AD in dogs have been reported worldwide, especially in areas where SuHV-1 circulates among domestic/feral pigs and wild boars [6, 28–40]. Most reports of AD concern dogs used for hunting wild boar and / or feral pigs [6, 32, 36, 38]. In fact, hunting exposes this category of dogs to a greater risk of infection due to direct and indirect contact with the reservoir population. The most common route of direct contagion is the ingestion of viscera and / or infected raw meat of wild boar and / or pigs [28, 33–35, 38, 40]. However, cases of AD following inhalation or penetration of the virus through traumatic wounds during hunting are also reported [34]. There are rare AD reports in dogs following contact with other infected species, such as badgers [31]. The risk of disease onset following exposure to attenuated SuHV-1 viral strains used in commercial vaccines for AD prophylaxis in pigs has also recently been reported [41, 42]. Neurological symptoms characterize the clinical picture of AD in dogs, often accompanied by intense neuropathic itching, which appears between 1 and 9–10 days from exposure to the virus [18, 31, 38]. Usually, itching might be diffuse or localized, reflecting the penetration site of the virus [18]. The suspicion of AD in dogs can be formulated based on a clinical history compatible with direct and/or indirect contacts with pigs and wild boars and on the presence of neurological symptoms accompanied or not by intense itching. The serological diagnosis, as performed in pigs by seroneutralization and ELISA (Enzyme-Linked immunosorbent assay), is not used in non-natural hosts, as the evolution of the disease is rapid, and death often occurs before antibodies are produced [43]. Therefore, diagnostic confirmation is obtained *post-mortem* by directly identifying the virus within the nervous system by viral isolation and molecular techniques (PCR and Real-Time PCR). The present study reports the first clinical AD cases in two dogs used for wild boar hunting in the province of Messina (Sicily), in an area close to the Nebrodi Natural Park, where AD cases have been reported in cattle [14]. These cases, even if sporadic, indicates that AD is present in the wild-swine population. This study represents the first well-documented report in this species in Sicily. The clinical, pathological aspects are described and the phylogenetic analysis of the SuHV-1 strain isolated is provided.

## Case presentation

**Fig. 1:**
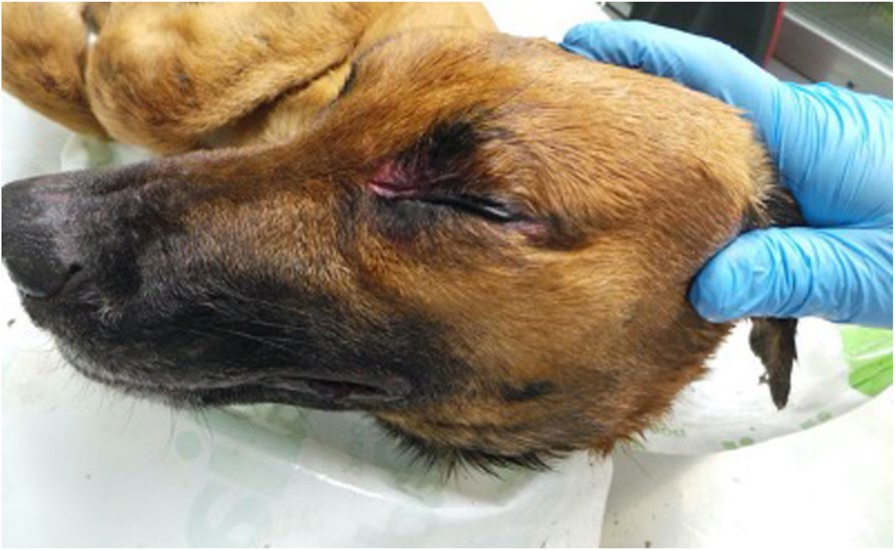
Dog 1: Injuries of traumatic origin due to intense itching in the peri-ocular region

**Fig. 2:**
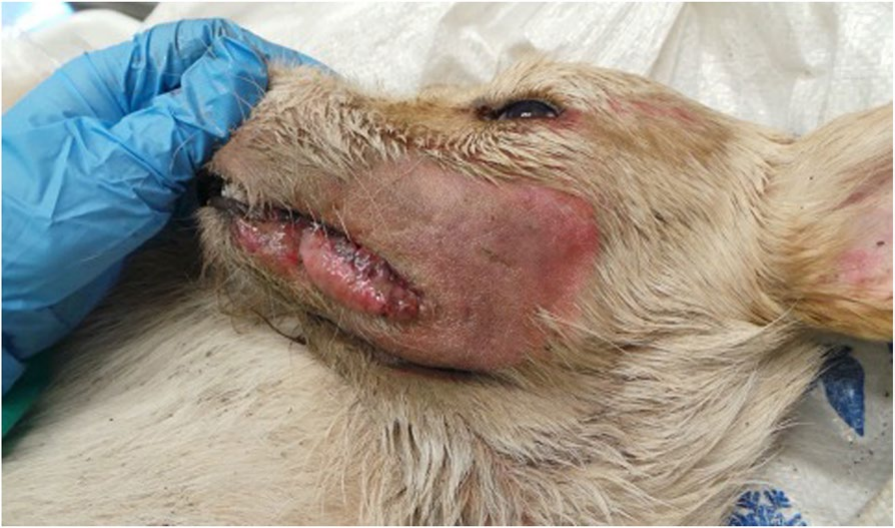
Dog 2: Injuries of traumatic origin due to intense itching in the peri-ocular region, cheek and labial region

**Fig. 3:**
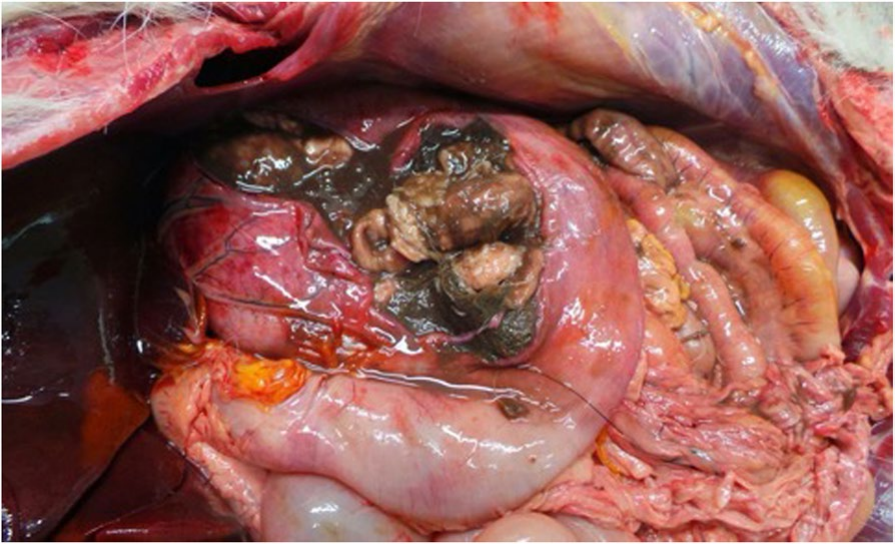
Dog 2: Examination of the splanchnic cavities and inspection of stomach contents. Highlighting of the presence of wild boar meat and offal

**Fig. 4:**
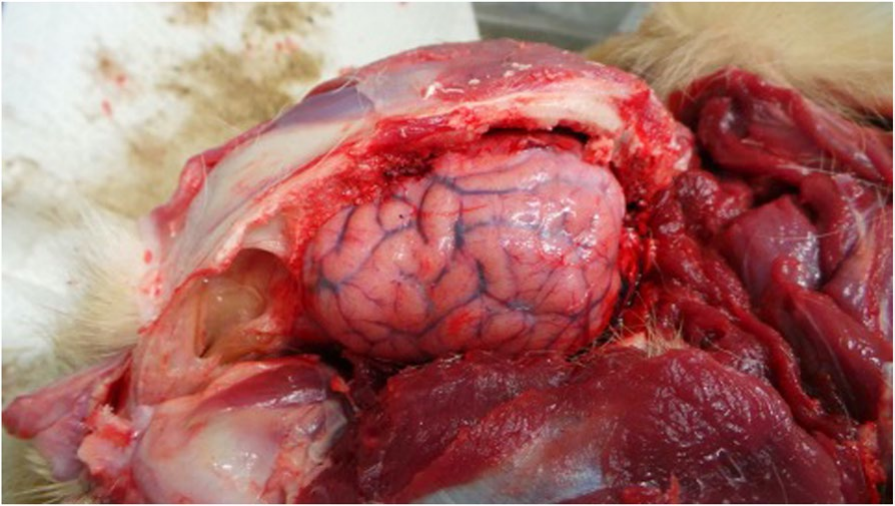
Dog 2: Evidence of hyperemia affecting the meninges and meningeal vessels

**Fig. 5:**
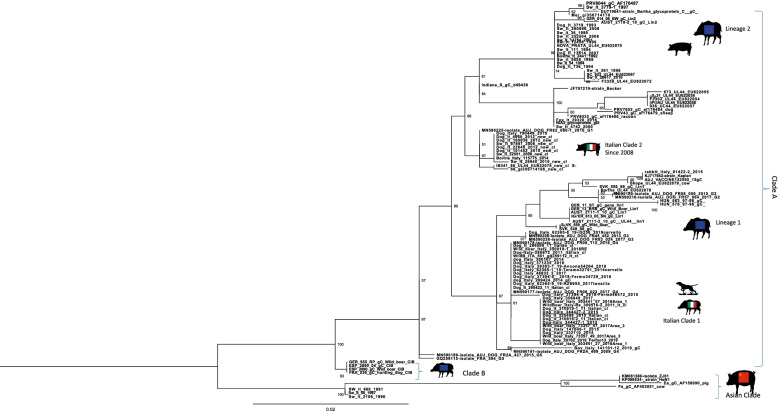
Phylogenetic tree based on partial sequencing of the UL44 gene. The tree was obtained using the maximum likelihood method and the HKY85 + I + G model with 1000 bootstrap replicates. The bootstrap percentage values are indicated at nodes. The Italian sequences are underlined

**Fig. 6:**
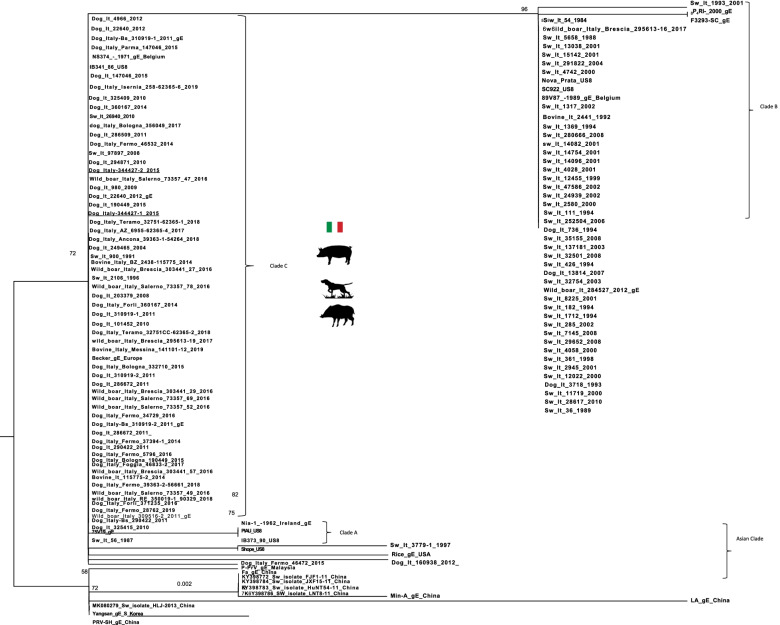
Phylogenetic tree based on partial sequencing of the US8 gene. The tree was obtained using the maximum likelihood method and the HKY85 + I + G model with 1000 bootstrap replicates. The bootstrap percentage values are indicated at nodes. The Italian sequences are underlined

The AD diagnosis was performed on two 4 years old mixed breed dogs, weighing around 20 kg, one male (Dog 1) and one female (Dog 2). The owner reported that both dogs were led hunting in the early hours of the morning in a large area adjacent to the Nebrodi Natural Park. The area is known for its high density of feral pigs and wild boars. No other dogs were involved in the hunting day. During the hunt, both dogs contracted repeated direct and indirect contact with wild boars only in the approach phase, a typical phase in which once the wild boar has been traced, the dog chases and leads the prey towards the place where the hunter places himslef in order to facilitate the kill. At the end of the hunt, culminating in the killing of a wild boar, on the same day, both dogs were given offal and viscera from the same hunted animal. Both individuals showed no significant symptoms in the following hours of the same day, appetite was preserved, and general behaviour was normal, apart from a small lacerated bruised wound near the lip margin showed by Dog 1. Clinical symptoms were evident 24 h and 72 h after the hunt for Dog 1 and Dog 2, respectively. Both dogs presented the same clinical picture and thus will be described together. Incoercible itching in the labial region and the neck, non-responsive to corticosteroid treatment, dominated the clinical picture reported by the referring veterinarian. Self-traumatic lesions due to the extreme itching were evident. Inspection of the buccal and ocular mucous membranes revealed massive congestion. The body temperature progressively increased until reaching values of 42° C in the sub-terminal stages. Both dogs showed sialorrhea and an increase in respiratory and heart rate. Neurological alterations were also observed with progressive depression, prostration and unresponsiveness to external stimuli. At intermittent intervals, the dogs manifested tonic–clonic contractions involving the main muscle groups. Both dogs died 48 h after the onset of clinical symptoms. The carcasses were referred to the Istituto Zooprofilattico Sperimentale della Sicilia (IZSS) – Area Barcellona Pozzo di Gotto (Messina), to perform post-mortem diagnostic investigations. Both carcasses were subjected to necropsy according to the internal procedures of the IZSS. The inspection was performed on skin and skin appendages, body cavities, splanchnic organs, central and peripheral nervous system. Both carcasses had a good nutrition state at the macroscopic examination with a Body Condition Score of 3 (scale of 1–5). Inspection of the skin and skin appendages did not reveal any significant alterations except for the presence of alopecic, hyperemic and erosive lesions present at the periocular and labial regions found in Dog 1 and 2, respectively (Figs. 1 and 2). In Dog 2, the lesions affecting the labial region were traumatic and lacerated-contused, extending to the skin overlying the masseters and appearing as a large hyperemic alopecic area (Fig. 2). The explorable mucous membranes (oculo-conjunctival and buccal) were hyperemic and congested. Inspection of the thoracic and abdominal cavities and the viscera revealed no significant alterations, except for muscle fragments and wild boar offal in the stomach (Fig. 3). Inspection of the brain, spinal cord and peripheral nerves showed alterations in the meningeal vessels of the telencephalon and especially the brainstem with the evident presence of hyperemia and congestion (Fig. 4). During the necropsy, samples of the brain, liver, kidney, spleen and cerebrospinal fluid were collected from both dogs and frozen at -80° C until virologic investigations. At the virology laboratories of the IZSS—Palermo area, the samples collected were processed and subjected to Real-time PCR for SuHV-1, as described by Yoon et. al 2005 [44]. The Real-Time PCR positive samples were then submitted to isolation on rabbit kidney cell lines (RK13) and porcine kidney cell lines (PK15) according to the standard operating procedures of the IZSS. All the samples were also subjected to Real-Time PCR for Rabies virus to exclude the disease as a differential diagnosis, thus resulting negative. Real-time PCR for SuHV-1 resulted positive in brain samples from both dogs (sample 344,427–1 from Dog 1 and sample 344,427–2 from Dog 2). Viral isolation on PK15 and RK13 cell lines was achieved only for sample n. 344,427–1, and immunofluorescence and Real-Time PCR were used as additional confirmatory tests for the presence of the virus. Molecular typing and sequencing of the Real-Time PCR products were performed at the National Reference Center for Aujeszky's Disease, Istituto Zooprofilattico Sperimentale della Lombardia e dell’Emila Romagna (IZSLER). Partial sequencing of the UL44 and US8 genes of two positive samples was performed, as previously described [6]. The sequences were edited using the SeqMan program (DNASTAR, Madison®, USA) and were compared to reference sequences and wild-type SuHV-1 strains available in GenBank for phylogenetic analysis. Phylogenetic trees were constructed using the maximum likelihood (ML) method within the IQ-tree software [45] with bootstrap analyses involving 1000 replicates. The sequence alignments were performed using the ClustalW method (DNASTAR, Madison, USA) and were manually optimized. The best-fit model of the nucleotide substitution was determined using the jModelTest v.0.1.1 [46]. All the models were compared using the Akaike’s information criterion (AIC) and the Bayesian information criterion (BIC). The preferred model was the HKY85 + I + G model. The topologies were verified with the neighbour-joining method and the Kimura two-parameter model using MEGA 7 [47]. Blast analysis of the gC sequences showed the highest identity rates (100%) with two Italian sequences, wild boar/Italy/309516/2/2011 [6] and dog/Italy/325409/2010 [48]. The first came from a wild boar in the Alps (Northern Italy) and the second from a hunting dog in the province of Bologna, both from locations far from the geographic origin of the canine sequences analyzed in this study. The phylogenetic tree of the UL44 genes, which is one of the most variable genes, showed three clades: A, B, and Asian (Fig. 5). The Italian strains all belong to the A clade except for three strains isolated in the 1990s that belong to the Asian clade [6]. The sequences studied here were placed in the Italian Clade 1 and wild boars and hunting dogs from Italy and France [6, 49]. The Italian gC sequences obtained from pigs and farm dogs formed the Italian clade 2 [6]. Italian strains belonging to Italian clades 1 and 2 showed different amino acid changes in the gC protein, which are highlighted in supplementary figure n.1. The sequences of the UL44 gene obtained in the present study from Dog 1 and Dog 2 were submitted in GenBank (accession number OL960553 and OL960554, respectively). The US8 gene encoding the gE protein was found to be a very conserved gene and, therefore, much less informative than the UL44 gene. Thus, the low number of information sites has led to a phylogenetic tree with not very high bootstrap values. Blast analysis of the two gE sequences showed 100% identity with most of the Italian wild boar, dog and pig samples whose gC sequences belonged to both Italian clades 1 and 2 [6]. Phylogenetic analysis of the US8 gene (Fig. 6) revealed the presence of 4 clades, named A, B, C and Asia, as reported in the study of Fonseca et al. (2010) [50]. The sequences of the two dogs were placed in the C clade together with all the Italian sequences that formed the Italian clades 1 and 2 in the UL44 phylogenetic tree. Interestingly, this clade was reported by Fonseca et al. (2010) [50] as a new clade that included only the strain IB341/86 (genBank acc number EU623990) isolated in Brazil in 1986. The sequences of the US8 gene obtained in the present study from Dog 1 and Dog 2 were submitted in GenBank (accession number OL960551 and OL960552, respectively). The sequencing of the complete genome of some samples characterized by gC sequences belonging to both Italian clades 1 and 2 should be performed to investigate better the different positions of the gC and gE sequences in their phylogenetic trees.

## Discussion and conclusions

The use of the dog for hunting big game is a traditional activity of great importance in Sicily. Hunting is mainly practised in those areas of the Region where the presence of large, wooded areas, undergrowth and the natural presence of food (berries, acorns, etc.) and watering sources has led over the years to a significant increase in the population of wild swine. The cases described occurred in province of Messina (northeast of Sicily). In Sicily is reared the 0.83% of the Italian pig population (8.900.328 animals—data updated as of 30/06/21—BDN Anagafe Nazionale Zootecnica) and 47.9% of Sicilian pigs are raised in free-range systems. More than half of free-roaming pigs (58%) are bred in the province of Messina, where the two cases described in this study are contextualized and where AD prevalence in farmed pigs is higher than in other Sicilian provinces [14]. Over the years, this type of management has favoured the uncontrolled increase of the wild swine population (wild boars, feral pigs, half-breed of pigs and wild boars), which is not included in the specific AD surveillance and control activities. However, the finding of AD in Sicily, in non-natural hosts such as cattle [14], foxes, sheep, goats, dogs and cats [51], although occasional, suggests that the disease is widespread in the Sicilian sylvan environment. This is probably due to the prevalent open-air pig farm system where appropriate biosecurity measures are difficult to apply in a rural context where direct and indirect contact with wild boars and wild pigs is often unavoidable. Furthermore, given the high prevalence of SuHV-1 in Sicily (6.48%) [52] and specifically in the province of Messina (12.20% in the period 2010–2019) [14], clinical cases of AD in hunting dogs are rarely reported in Sicily, and the authors believe that AD in species other than swine is underestimated. Thus, the sporadic nature of the disease leads to the reporting only of only striking cases. However, it is not uncommon to receive post-hoc informal reports of suspicion of AD in dogs used to hunt feral pigs and wild boar from freelance veterinarians, which therefore remain undiagnosed. The cases reported in this study, in fact, represent a rare opportunity to make a diagnosis since the carcasses of the two dogs were reported directly in the IZSS laboratories and are actually the first well-documented AD reports in hunting dogs in Sicily. Hunting dogs are notoriously exposed to the risk of AD contagion since during hunting trips, close contact with wild boars and feral pigs and the common practice of feeding hunting dogs with raw meat and offal from hunted animals greatly increases the risk of AD transmission between species. In our cases, the first suspicion of the AD was based on the anamnestic information of the previous direct contact with wild boar, on the detection of ingested offal and raw wild boar meat as well as on the presence of uncontrollable itching, unresponsive to corticosteroid therapy, and non-specific neurological symptoms. The symptomatic picture described in the presented cases is comparable to what has already been documented in various case reports of AD in dogs [6, 31–40]. The predominant symptom is intense and incoercible itching, classified as "neuropathic pruritus", which is considered to be a typical sign of AD in hosts other than swine [18, 29, 46, 53]. Only infrequent reports describe cases of AD in dogs without pruritus [30]. In the two cases reported, the itch was restricted to the head and neck region in both cases. In most of the reports, these regions seem to be the most frequently involved [32, 37, 38, 40], except for sporadic cases in which the itchy areas were located in the posterior region of the body [34]. Experimental studies on the pathogenesis of AD in dogs [18] and other non-natural hosts [54] suggest that the different localization of pruritus is related to the pathway of virus penetration. The Head and the neck are mainly involved when infection occurs via the oral and / or respiratory mucosa [18, 54, 55]. In our case, the infection likely arose through the oral mucosal and/or by inhalation of viral particles during the ingestion of wild boar raw viscera, which represents the direct route of contagion more common in hunting dogs [28, 33–35, 38, 40]. However, it is not possible to exclude that the infection also occurred during the direct and indirect contacts that the dogs established approaching the wild boar. While the presence of itching facilitates the clinical diagnosis of AD in dogs, other potential causes of itching, such as parasitic infestations and bacterial and viral infections, must be excluded in the diagnostic process. In our case, the inspection of the skin and skin appendages excluded this possibility. However, the main disease for which a differential diagnosis is mandatory, is Rabies, as it causes similar neurological alterations and aggressive attitude. In our case, the results of the Real-Time PCR of the samples of the brain, spleen, kidney and liver excluded this diagnosis. However, it is useful to report the presence of AD cases in dogs that can occur in the absence of itching, and in the latter case, the differential-diagnostic process must necessarily be broader [33]. In the present cases, no specific macroscopic lesions were detected, except for self-traumatic lesions due to intense itching and non-specific lesions at the level of the Central Nervous System (hyperemia and injection of the meningeal vessels, especially at the level of the telencephalon and the brainstem), as already reported [30]. The absence of pathognomonic lesions is not surprising as it is a characteristic of AD in hosts other than swine species [9]. The confirmation of AD was obtained based on the positivity found for SuHV-1 at Real-Time PCR in both brains of the two animals and the virus isolation from the two brain samples. The central nervous system represents the most common matrix for diagnostic molecular methods for viral identification and isolation [6, 28–40]. The greater probability of finding the virus at this level depends on the pathogenetic progression of the disease. After penetration at the site of infection, the virus spreads from the peripheral nervous system up to the central nervous system, using centripetal axonal transport. The infection results in a non-suppurative inflammation, causing irreversible damage that leads the animal to death [18, 33]. The SuHV-1 strains isolated from the two dogs and the sequencing data obtained enriches the gC phylogenetic tree with sequences unknown to date and therefore not listed in the GenBanK. The phylogenetic analysis of the isolated strains showed a high similarity with strains isolated in wild boars and recently in cattle [14], grouped in the Italian Clade 1 (Fig. 5). These two cases occurred in a large rural area that includes the Nebrodi natural park, which is peculiar for the presence of a typically multi-host complex livestock system consisting of prevalent mixed, free-range and transhumant farms that have common grazing areas with the invasive wild swine population. The similarity of the strains isolated from cattle and dogs leads to the hypothesis that there is a common source of sylvan exposure and that it is not possible to exclude that it is the cause that hinders an effective control of AD in pigs reared in Sicily. Although multiple human and financial resources have focused on AD control, the disease is still prevalent in Sicily (6.48%) [52]. Only recently, the Sicily Region (Circular Health Department protocol 00 21,810 of 11–06- 2020), have applied more stringent AD control measures and a regional control plan for the eradication of AD (annex II of 2008/185 / EC—version of 2021–03-04). However, a specific surveillance activity for wild swine is not foreseen, and the absence of data on the spread of AD does not allow us to date, to evaluate the potential risk of interspecies transmission in non-natural hosts, as well as the potential threat to disease eradication in free-range pig farms. The cases presented in this study, together with the other Sicilian reports in other species (dogs, cats, sheep, goats, cattle and foxes) [14, 51], suggest that SuHV-1 is widespread in a sylvatic environment and that there is a potential risk of SuHV-1 exposure to other susceptible species. Particular attention should be paid to hunting dogs, whose contagion risk is greater than other species. Future studies should be conducted in these areas to obtain information about the circulation of SuHV-1 strains to better target AD control strategies.

Finally, a training and information campaign aimed at hunters and freelance veterinarians should be activated, allowing the creation of a virtuous communication network based on the diagnosis of suspected cases and prevention.

## Supplementary Information

**Additional file 1:Figure 1.** Amino acid sequences of the gC protein of Italian dog samples belonging to the Italian clades 1 and 2.

## Data Availability

The sequences obtained in the current study are available at genBank, accession numbers OL960551, OL960552, OL960553 and OL960554.

## Abbreviations

AD: Aujeszky's disease
SuHV-1: Suid Herpes Virus 1
ELISA: Enzyme-Linked immunosorbent assay
IZSS: Istituto Zooprofilattico Sperimentale della Sicilia
IZSLER: Istituto Zooprofilattico Sperimentale della Lombardia e Emilia Romagna
