# Supplementary figures and images for: Aujeszky’s disease in hunting dogs after the ingestion of wild boar raw meat in Sicily (Italy): clinical, diagnostic and phylogenetic features
Source: BMC Vet Res. 2022 Jan 8;18:27. doi: 10.1186/s12917-022-03138-2 (PMC8742332; doi:10.1186/s12917-022-03138-2)

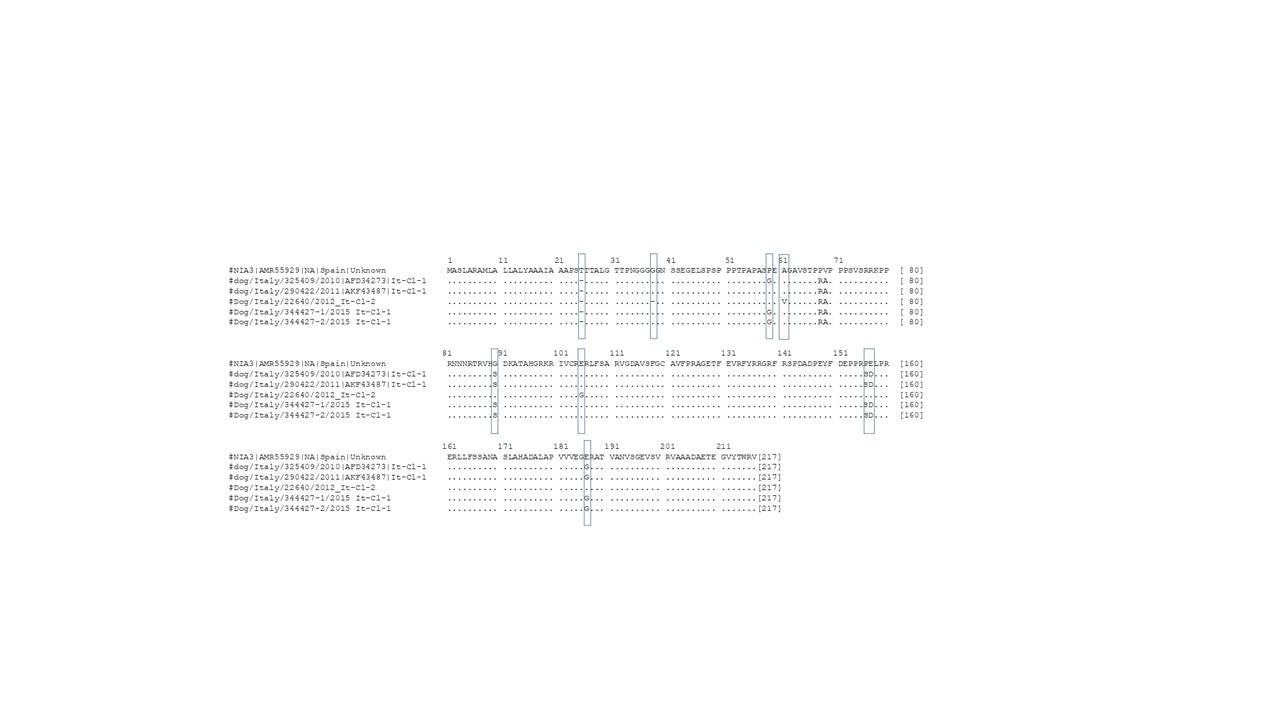

Supplement: Supplementary file 1 — Additional file 1:Figure 1. Amino acid sequences of the gC protein of Italian dog samples belonging to the Italian clades 1 and 2. [file 12917_2022_3138_MOESM1_ESM.jpg]
